# The clinical usefulness of knowing *CHRNA5* polymorphism genotype: paving the way for personalized therapy

**DOI:** 10.1177/03008916251408279

**Published:** 2026-03-10

**Authors:** Francesca Colombo, Chiara Veronese, Elena Munarini, Cinzia Paolino, Davide Maspero, Nunzia Mangano, Martina Esposito, Francesca Minnai, Sara Noci, Marta Giussani, Daniele Morelli, Elisa Cardani, Alessandro Esposito, Gaia Giulia Angela Sacco, Debora Spitaleri, Roberto Boffi

**Affiliations:** 1 Department of Research, Fondazione IRCCS Istituto Nazionale dei Tumori, Milan, Italy; 2 National Research Council, Institute for Biomedical Technologies, Segrate, Italy; 3 Department of Advanced Diagnostics and Services, Pulmonology Unit, Fondazione IRCCS Istituto Nazionale dei Tumori, Milan, Italy; 4 Department of Experimental Oncology, Fondazione IRCCS Istituto Nazionale dei Tumori, Milan, Italy; 5 Department of Advanced Diagnostics and Services, SC Laboratory Medicine, Fondazione IRCCS Istituto Nazionale dei Tumori, Milan, Italy

**Keywords:** epidemiology and prevention, smoking cessation, pharmacogenomics

## Abstract

**Background:**

Tobacco smoking is a leading cause of global mortality, with cessation being the primary prevention strategy. Nicotine addiction has a genetic component; the rs503464 single nucleotide polymorphism (SNP) in the *CHRNA5* gene is associated with smoking cessation therapy success. However, the impact of communicating genetic risk to patients remains unclear. This study evaluated whether knowledge of the rs503464 genotype influences smoking cessation rates.

**Methods:**

270 smokers were enrolled and randomized into two groups: informed and uninformed of their rs503464 genotype. All participants received standard pharmacological-behavioral interventions. Cessation rates were assessed at 1, 3, 6, and 12 months. Multivariable logistic regression models analyzed the effect of knowing the rs503464 genotype and other variables on cessation success.

**Results:**

Among the 219 subjects who started prescribed smoking cessation medication, no significant differences in cessation rates were observed between participants informed or not informed of their rs503464 genotype at any follow-up point (P > 0.05). Male gender and higher baseline carbon monoxide levels were associated with lower success rates at three months. The medications used were equally effective.

**Conclusions:**

Communication of the rs503464 genotype did not influence smoking cessation success, proving that it does not disturb this process. This result opens the possibility of using genetic information to personalize anti-smoking treatment.

## Introduction

It is estimated that more than 8 million deaths attributable to tobacco use occur globally each year. One-third to one-half of people who regularly smoke cigarettes die from a tobacco-related disease, and generally about 10 years earlier than non-smokers.^1^ Quitting smoking is one of the primary prevention strategies against the development of significant types of chronic diseases, including those of the cardiovascular and respiratory systems and cancer.^2^ However, managing to quit smoking is very difficult, and the success rate without professional help is achieved in only 3-5% of cases.^3^ At the basis of this difficulty, there is nicotine and the related high affinity α4β2* nAChR receptors; these play a key role in the behavioral actions of nicotine that contribute to the development of tobacco dependence, including its effects on brain circuits involved in reinforcement.^4^

Twin studies have shown that nicotine addiction is, at least in part, genetically determined.^5,6^ Germline genetic variants in genes involved in nicotine binding (e.g., nicotinic acetylcholine receptor subunits) and metabolism (e.g., cytochrome enzymes) have been investigated for their association with this phenotype.^7^ Nicotine addiction is a complex polygenic phenotype, and thus far, at least 32 independent genetic loci have been reported to be associated with this trait in several studies, as reported in the genome-wide metanalysis by Hatoum et al.^8^

The most effective treatment to deal with tobacco addiction consists of a multimodal approach based on pharmacological therapy plus cognitive-behavioral support.^9^ First-line drugs have proven effective in treating tobacco addiction, have a higher safety level, and are approved by the European Medicines Agency (EMA). These therapies include nicotine replacement therapy, varenicline, and bupropion. For a decade varenicline has been considered as the reference treatment in smoking cessation but has been withdrawn from the market for safety reasons.^10^ Second-line drugs include cytisine, a nicotine receptor partial agonist. Although cytisine is still considered a second-line drug according to European guidelines,^9^ numerous studies demonstrate its safety and effectiveness.^11–14^

Some pharmacogenetic studies have investigated the association of germline genetic variants with the success/failure of smoking cessation therapies, sometimes reporting conflicting results.^15^ In our laboratory, we have previously identified a statistically significant association between the genotype of a single nucleotide polymorphism (SNP), rs503464, and the success of smoking cessation therapies.^16^ This variant is mapped in the 5' regulatory region of the *CHRNA5* gene. It is also associated with mRNA levels of this gene, which encodes the alpha-five subunit of the nicotinic acetylcholine receptor. We observed that individuals carrying at least one minor allele of this SNP had a lower risk of failure after smoking cessation treatments compared to patients homozygous for the major allele. This important discovery opens the scenario to the possibility of prescribing personalized pharmacological-behavioral therapies based on genetic data. However, before personalizing therapy, it is necessary to assess whether communicating genetic data on the risk of failure may or may not influence the outcome of the cessation process.

This consideration raises the pertinent issue of genetic data dissemination and the subsequent management thereof. While the communication of genetic risk has been extensively investigated across numerous medical domains, with the objectives, contexts, implications for clinical decision-making, and ethical ramifications of genetic counseling having been well-defined, the communication of genetic susceptibility specifically concerning smoking cessation presents a distinct paradigm. Unlike genetic predispositions to disease, this context involves an addictive behavior wherein individual motivation, alongside genetic factors, constitutes a significant determinant. Given the current paucity of established scientific literature addressing this specific intersection, the present study aims to elucidate the potential influence of communicating genetic data on the outcomes of smoking cessation endeavors.

## Materials and methods

### Study cohort and ethical statement

For this double-blind, randomized study, 270 smokers were enrolled at the Tobacco Control Unit of the Fondazione IRCCS Istituto Nazionale dei Tumori in a period spanning from October 2019 to September 2021. The recruitment was discontinued from March to July 2020 due to the COVID-19 pandemic. The Declaration of Helsinki was used to perform the study. The research was approved by the ethics committee of Fondazione IRCCS Istituto Nazionale dei Tumori, Milan, Italy (protocol number INT 126/19). All participants provided written informed consent to participate in the study, granting permission to use their biological samples and clinical data.

### Study design and procedures for clinical data and biological material collection

At enrollment, individuals underwent a routine medical examination provided by the Pulmonology Unit. This examination included personal and clinical data collection, in particular regarding subjects’ smoking status, such as the number of cigarettes per day (CPD), pack-years (P/Y), and the Fagerström test for nicotine dependence, pulmonary examination, and measurement of exhaled carbon monoxide (hereafter referred to as eCO). A peripheral blood sample was also collected in EDTA (Ethylenediaminetetraacetic acid) for genomic DNA extraction.

During this first medical examination, before knowing the data relative to polymorphism, the clinician prescribed one of the following smoking cessation drugs: varenicline, cytisine, nicotine replacement therapy, bupropion, or a combination of these latter two, following the best standards of care for dosage and treatment duration, used in clinical practice.

Then, the genomic DNA of each patient was extracted, and rs503464 was genotyped (see below for extraction and genotyping methods). Patients were randomized into two groups: one in which the individual’s genotype would be blinded to both the subject and the medical staff, and the other in which the genotype would be unblinded. To perform randomization, we devised a minimization program using the R package "Rand Pack" and based on Pocock's method^17^ to randomize smokers by minimizing the imbalance in the number of subjects in each group for several factors: sex, age, genotype of SNP rs503464, and type of medication prescribed.

**Figure 1.**
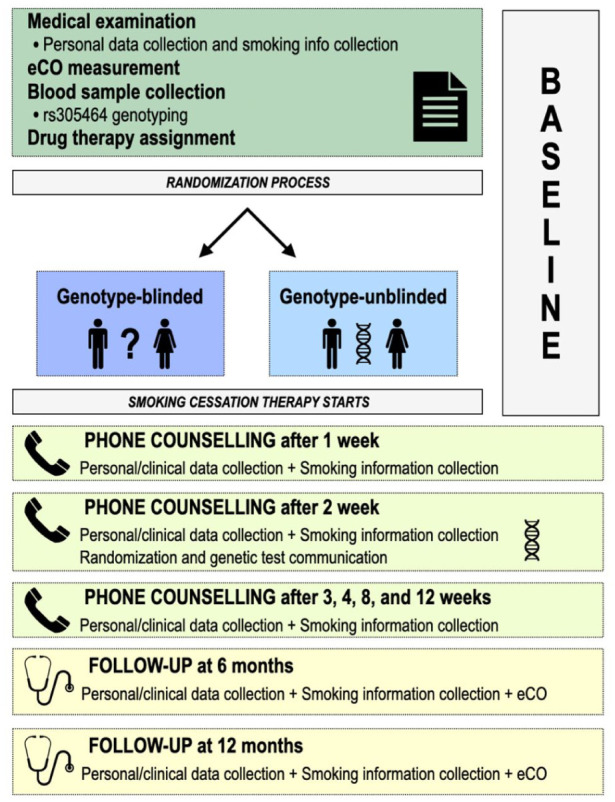
Study design.

After 1, 2, 3, 4, 8, and 12 weeks from the start of therapy, individuals received telephone counseling sessions from the psychologists of the anti-smoking center. During the second phone call, two weeks after the beginning of treatment, enrolled patients were informed of their assigned arm, with the unblinded group also receiving their genetic risk information. To mitigate potential variability in the psychological impact of genetic risk disclosure, all participating clinical psychologists used a standardized text to communicate the rs503464 genotype information (Online Supplementary Material S1). This protocol ensured uniformity in the linguistic framing and delivery of genetic risk assessment to participants in the unblinded study arm. Subjects who did not start drug treatment were excluded from the study. Smoking abstinence was defined as seven-day point prevalence abstinence, based on self-reported smoking status at each follow-up timepoint. Continuous abstinence was not assessed in this study. At 1 and 3 months, data on response to therapy (cessation or not of smoking habit) and CPD were collected by phone call. Six months after the start of therapy, all patients were scheduled for a follow-up visit to collect data on response to treatment, CPD, and measurement of eCO. One year after the start of therapy, patients had a final follow-up visit with collection of response data, as done at earlier time points, and measurement of eCO level. The number of phone calls and medical checks was standard in both blinded and unblinded patients at low or high risk, so that the support received by the patient did not influence the outcome of the process. Figure 1 shows a representation of the study design.

### DNA extraction and genotyping

Genomic DNA was extracted from blood samples using the DNeasy Blood & Tissue kit (QIAGEN) and quantified using the NanoDrop 2000c UV–Vis spectrophotometer (Thermo Fisher Scientific). Genotyping of SNP rs503464 was performed using the C_9120_20 TaqMan genotyping assay (Thermo Fisher Scientific). The genotype of each subject was determined by allelic discrimination analysis conducted using TaqMan Genotyper software (Thermo Fisher Scientific).

### Statistical analyses

Chi-squared or Fisher's Exact tests were used to compare frequencies between groups. The Kruskal-Wallis test was used to compare medians of quantitative variables between groups. Comparisons between paired values of CPD (at baseline versus each of the four follow-up times) were carried out using the Wilcoxon paired test. Multivariable logistic regression models were performed to test the effect of personal and clinical variables (i.e., age, sex, administered drug, baseline smoking quantity measures, and genotype of rs503464 coded as in a dominant model) on the response to smoking cessation therapy, at each of the four follow-up times (1, 3, 6, and 12 months). This is the formula for the tested model:

$$\begin{array}{l} \mathrm{Response}\;\mathrm{at}\;N\;\mathrm{months}\;\sim \;\mathrm{sex}\;+\;\mathrm{age}\;+\;\mathrm{baseline}\;\mathrm{CPD}\\ \;\;\;\;\;\;\;\;\;\;\;\;\;\;\;\;\;\;\;\;\;\;\;\;\;\;\;\;\;\;\;\;\;\;\;\;\;\;\;\;\;\;\;\;\;\;\;\;\;\;+\;\mathrm{baseline}\mathrm{eCO}+\mathrm{base}\;\mathrm{line}\;\mathrm{pack}\\ \;\;\;\;\;\;\;\;\;\;\;\;\;\;\;\;\;\;\;\;\;\;\;\;\;\;\;\;\;\;\;\;\;\;\;\;\;\;\;\;\;\;\;\;\;\;\;\;\;\;-\mathrm{year}\;+\;\mathrm{baseline}\;\mathrm{Fagestr}öm\;\\ \;\;\;\;\;\;\;\;\;\;\;\;\;\;\;\;\;\;\;\;\;\;\;\;\;\;\;\;\;\;\;\;\;\;\;\;\;\;\;\;\;\;\;\;\;\;\;\;\;\;+\;\mathrm{drug}\;+\;\mathrm{genotype} \end{array}$$

where the administered drug was coded as three categories, the subjects treated with cytisine, varenicline, or other drugs (grouping together subjects treated with bupropion, nicotine replacement therapy, and combined therapy). Only the results of the multivariable models selected with a stepwise procedure based on Akaike information criterion (AIC) were reported.

Similarly, we tested the effect of knowing the rs503464 genotype, at each of the four follow-up times (1, 3, 6, and 12 months), in multivariable models with those variables passing AIC stepwise model selection, as covariates. A threshold for significance for all tests was set at *P*-value<0.05.

## Results

For this study, 270 smokers were enrolled at the Pulmonology Unit of the Fondazione IRCCS Istituto Nazionale dei Tumori, Milan (Italy). Among them, 219 started the prescribed smoking cessation medication, while 51 abandoned the anti-smoking program or preferred to tackle it without pharmacological support and were therefore excluded. Subjects' characteristics at baseline are summarized in Table 1. They were equally distributed between males and females; their median age was 56. On average, they smoked 20 cigarettes per day, and the median eCO value before smoking cessation therapy was 21 ppm. About half of them previously attempted to quit smoking at least once in their lifetime, but less than 10% used smoking cessation medication for this aim. The two drugs mainly prescribed in this study were cytisine and varenicline (with approximately the same frequency). In contrast, bupropion, NRT (nicotine replacement therapy), or a combination of them was administered to less than 20% of enrolled subjects. As expected from the minor allele frequency (MAF) of rs503464 SNP in Europeans (0.26 according to 1000 Genomes Project Phase 3), recruited individuals were mainly (58%) homozygous for the T allele; the MAF in our series was 0.23.

**Table 1.** Baseline personal and clinical characteristics of the enrolled subjects who started smoking cessation therapy.

Characteristic	Enrolled subjects (n = 219)	Group		Kruskal-Wallis or Fisher test *P*-value
		Blinded (n=112)	Unblinded (n=107)	
Sex, n (%)				0.89*
*male*	107 (49)	54 (48)	53 (50)	
*female*	112 (51)	58 (52)	54 (50)	
Age, median (range), years	56 (24-79)	58 (26-79)	56 (24-79)	0.78
CPD, median (range)	20 (2-80)	20 (4-80)	20 (2-50)	0.59
Pack-years, median (range)	33 (1-180)	32 (1-180)	33 (5-114)	0.88
eCO, median (range), ppm	21 (0-71)	21 (1-60)	21 (0-71)	0.90
Fagerstrom test, median (range)	5 (0-10)	5 (0-10)	5 (0-9)	0.27
Mondor test, median (range)	13 (2-20)	12.5 (5-18)	13 (2-20)	0.77
At least one previous attempt to quit smoking, n (%)	97 (44)	49 (44)	48 (45)	1.00
Smoking cessation drug assumption in previous attempts to quit smoking, n (%)	19 (9)	9 (8)	10 (9)	0.81
Drug, n (%)				0.38*°
*Bupropion*	15 (7)	9 (8)	6 (6)	
*Cytisine*	99 (45)	47 (42)	52 (49)	
*Nicotine replacement therapy*	17 (8)	11 (10)	6 (6)	
*Varenicline*	84 (38)	43 (38)	41 (38)	
*Combined*	4 (2)	2 (2)	2 (2)	
rs503464 genotype, n (%)				0.89*^
*AA*	9 (4)	2 (2)	7 (7)	
*AT*	82 (37)	44 (39)	38 (36)	
*TT*	128 (58)	66 (59)	62 (58)	

CPD, cigarettes per day; eCO, exhaled carbon monoxide; SD, standard deviation. ° The three less numerous types of treatments (bupropion, nicotine replacement therapy and combined therapy) were grouped together and compared with cytisine and varenicline groups. ^ AA and AT genotypes compared with TT genotype. * Fisher test *P*-value

As expected from randomization, no significant differences were observed between the individuals in the two experimental arms of the study (blinded and unblinded for rs503464 genotype) in terms of age, sex, Fagerström and Mondor test scores, history of previous quit attempts, previous use of cessation drugs, MAF, and present therapies (chi-squared and Kruskal-Wallis test *P*-values > 0.05). Additionally, there were no significant differences (Kruskal-Wallis test *P*-values > 0.05) between the two groups regarding CPD, pack-years, and eCO.

**Figure 2.**
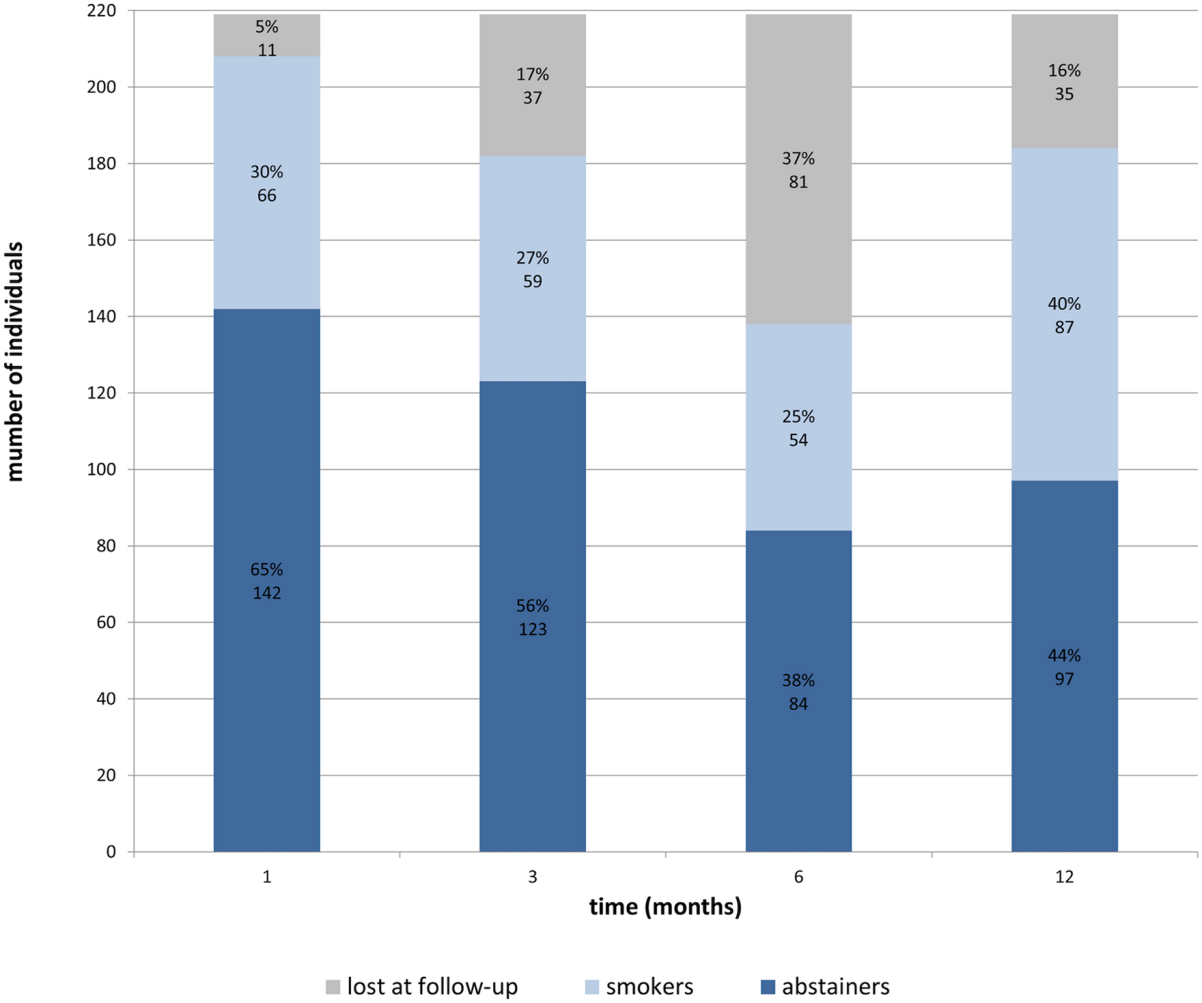
Response to therapy at the four follow-up times. The labels show the percentages and number of individuals in each group at different times.

Data on treatment response were collected 1, 3, 6, and 12 months after smoking cessation therapy started (Figure 2). In detail, 65% (142/219) of patients undergoing drug therapy were abstinent for one month from the start of treatment. After three months, the percentage of abstainers dropped to 56% (123/219) and then fell further, at 6 (38%, 84/219) and 12 months (44%, 97/219). As previously reported,^16^ the success of smoking cessation therapies decreases with time. Notably, the percentage of patients lost at follow-up ranged from 5% to 17%, except at 6 months, when the expected information could not be collected for more than a third of the patients (37%). This data loss occurred because of the pandemic and restrictions on activities during the lockdown period in Italy. About 70 patients had been enrolled between the end of 2019 and March 2020, so the 6-month time point occurred during the lockdown months or shortly thereafter, preventing the planned visits.

**Figure 3.**
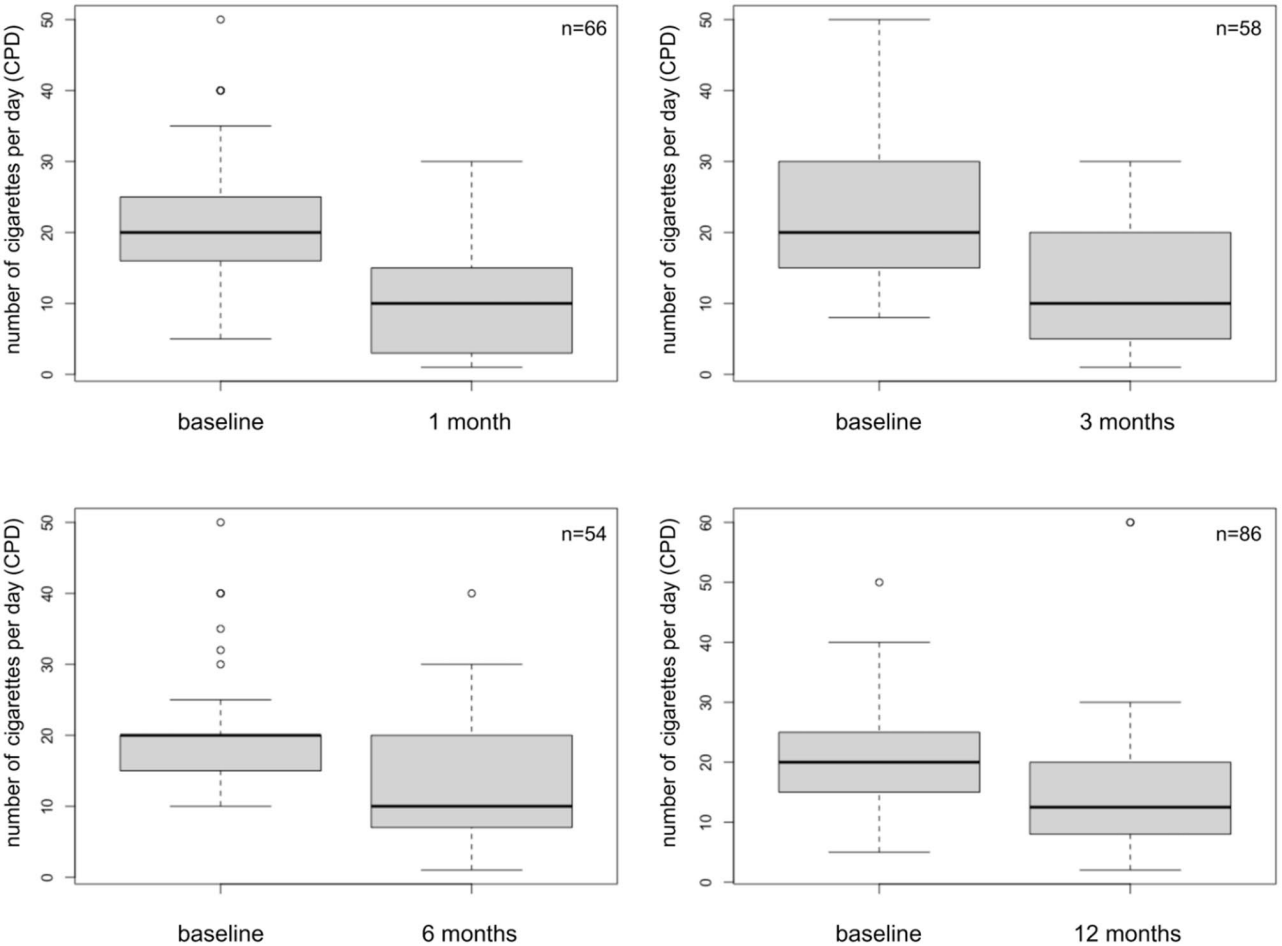
The number of cigarettes smoked per day by individuals who remained smokers was lower than baseline at all four time points (P-value < 0.05, Wilcoxon test). The number of subjects analyzed is shown.

One year after the start of smoking cessation therapy, 41 subjects remained smokers, and 46 experienced at least a relapse during the anti-smoking journey. Among the 87 individuals who failed to quit, the median CPD was 10 (range 1-30, at 1 and 3 months; range 1-40, at 6 months) in the first 6 months and 12.5 (range 2-60) one year after the start of therapy. At any time point, smokers had lower CPD than the reported values at the baseline (Wilcoxon test *P*-value <0.05; Figure 3), thus indicating that although the smoking cessation therapy failed for these individuals, at least it contributed to reducing CPD.

Using multivariable logistic models, we evaluated the effect on the response to anti-smoking therapy of the different medications administered, sex, age, and baseline smoke quantity measures (CPD, pack-years, eCO, Fagerström test result), and rs503464 genotype, at the four different time points. Of note, no significant differences were observed in the success rate according to the administered drug (comparisons were made between subjects treated with cytisine, varenicline, and the other drugs pooled together due to the small number of subjects in the other groups) at any time point. This observation indicates that the different medications used in the study were equally effective (Online Supplementary Material S2). We observed that the baseline eCO, was associated with the response to the smoking cessation therapy at 1 and 3 months: in detail, individuals with a high eCO value at the baseline had a lower probability of quitting smoking (OR=0.97, *P*-value=0.013; OR=0.96, *P*-value=0.0050; Table 2).

**Table 2.** Independent variables affecting the response to smoking cessation therapy at 1, 3, 6, and 12 months in multivariable logistic models resulted from stepwise model selection based on AIC (OR>1 indicated a higher probability of success in quitting smoking).

	OR	95% CI	*P*-value
*1 month*			
age	0.97	0.95 – 1.0	0.057
eCO	0.97	0.94 – 0.99	**0.013**
genotype (AA+AT)	1.7	0.91 – 3.2	0.10
*3 months*			
age	0.97	0.94 – 1.0	**0.038**
sex (male)	0.49	0.25 – 0.96	**0.039**
eCO	0.96	0.94 – 0.99	**0.0050**
genotype (AA+AT)	2.00	1.0 – 4.1	**0.050**
*6 months*			
eCO	0.97	0.94 – 1.0	0.064
genotype (AA+AT)	3.4	1.6 – 7.7	**0.0021**
*12 months*			
eCO	0.98	0.96 – 1.0	0.098

OR, odds ratio; CI, confidential interval; eCO, exhaled carbon monoxide.

Three months after the start of therapy, significant differences in the response between males and females were observed, with a lower probability of success for males (OR=0.49, *P*-value=0.039, Table 2). Also, the individuals with AA or AT genotypes at rs503464 significantly differ from those homozygous for the major allele (GG) in the response to therapy at 3 and 6 months: in detail, they more likely succeeded in quitting smoking (3 months: OR=2.0, *P*-value=0.0496; 6 months: OR=3.4, *P*-value=0.0021 Table 2). However, neither of these associations was statistically significant at 12 months.

**Figure 4.**
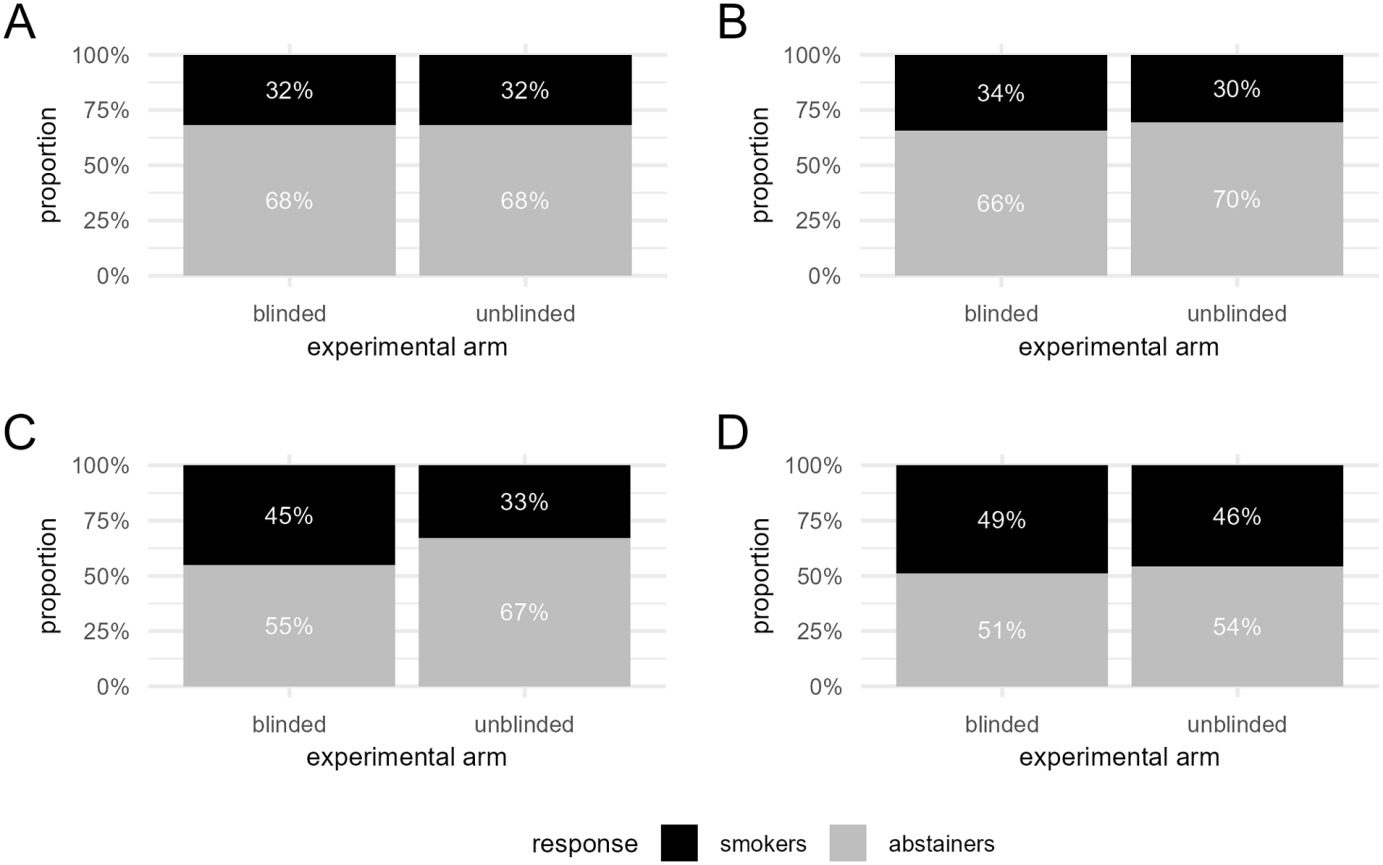
Knowing the genotype of rs503464 did not significantly affect the success rate of smoking cessation therapy at (A) 1 month, (B) 3, (C) 6, and (D) 12 months.

We did not observe any significant difference in the frequency of individuals who stopped smoking versus those who did not stop between the two experimental arms (i.e., blinded and unblinded for the rs503464 genotype; chi-squared test *P*-value > 0.05, Figure 4), at any of the four times of follow-up, thus indicating that knowing the genotype of rs503464 does not affect the success rate of the smoking cessation therapy.

We also tested the effect of knowing or not knowing the rs503464 genotype on the smoking cessation therapy response in multivariable logistic regression models (at the four different time points), with variables passing Akaike stepwise model selection reported above, as covariates. No significant differences, at any time point, were observed between blinded and unblinded individuals in terms of smoking cessation therapy response, also in these models adjusted for the other variables (Online Supplementary Material S3).

## Discussion

Precision medicine, also known as "personalized medicine," is an innovative approach to tailoring disease prevention and treatment that considers differences in people's genes, environments, and life. Understanding human genetic variation and its impact on gene function is an essential goal in genomics-based research. Pharmacogenetics/pharmacogenomics exemplifies the translation of this knowledge into clinical care. Identifying particular gene variants that may influence drug kinetics and dynamics allows for a more effective personalized medicine approach in choosing the right drug or dose for a specific individual.^18^

Although personalized medicine is now widespread in many fields, it has not fully reached smoking cessation therapies and research on how sharing genetic risk affects patient motivation and quitting smoking is limited. Some recent studies have highlighted the growing potential of pharmacogenetics to personalize smoking cessation treatment by identifying responders to varenicline or nicotine replacement therapy based on genetic variants in *CHRNA5* and *CYP2A6*.^19,20^ We previously identified a statistically significant association between the genotype of a single nucleotide polymorphism (SNP), rs503464, and the success of smoking cessation therapies^16^; however, proceeding with the personalization of treatment, based on the detected genetic risk, requires some methodological steps to move safely for the benefit of patients.

This study focuses on the first of these steps: communicating the genetic data. A crucial aspect to consider in the clinical application of a genetic test predictive of smoking cessation success is its potential psychological impact. The success of an attempt to quit smoking largely depends on the motivational structure of the person that the smoking cessation expert must support and strengthen. Genetic risk information must not discourage the current motivation enough to lead to abandoning the attempt to quit. This risk is, in fact, different from other types of genetic risk in which knowing that there is a genetic risk can be a source of anxiety, but does not harm the behavior. After receiving information about genetic risk, a person may decide not to change his life based on this information, or to do, by undergoing more frequent checks, preventive protocols, or by changing his lifestyle. On a psychological level, the information on the genetic risk of succeeding or failing to quit smoking could be the cause of counterproductive reactions in both outcomes. If the result of the genetic test is that there is a greater chance of being able to quit, this can lower determination and commitment, and vice versa; if the result is of more incredible difficulty, it can lead to a reduction in self-confidence and, similarly to the first case, less commitment.

Our results demonstrate the absence of negative sequelae associated with disclosing genetic data after treatment initiation. Building upon this, the next phase of our research will incorporate genetic information into the parameters guiding treatment decisions. Furthermore, future studies will aim to evaluate the efficacy of augmented interventions, addressing both pharmacological and psychological dimensions (in quantity, duration, and intensity), specifically for individuals identified as having an increased genetic predisposition to unsuccessful smoking cessation.

The findings of this study should be interpreted in light of the limitations, the foremost being its exploratory nature. The limited number of participants (only 270 enrolled initially, with 219 starting medications, and even fewer at certain time points, due to loss to follow-up) may have resulted in insufficient statistical power to detect a significant effect of genotype knowledge on smoking cessation rates, if such an effect exists. A low statistical power also can result in missing small or moderate effects and tends to produce results that are less reliable and difficult to replicate. The study's exclusive focus on the rs503464 single nucleotide polymorphism (SNP) in the *CHRNA5* gene might be an oversimplification of the complex genetic architecture underlying nicotine addiction and smoking cessation success. Multiple genes and their interactions are likely to contribute to this trait, and examining only one variant may not capture the full genetic influence. Furthermore, the effectiveness of the genotype communication in motivation, perception of risk, or adherence to the cessation program could have been influenced by how the information was presented and understood by participants, which could have impacted the study's findings. It is possible that a more tailored or impactful method of communicating genetic risk could yield different results. In addition, as smoking status at 1 and 3 months was assessed through telephone interviews without biochemical validation, potential self-reporting bias cannot be excluded. Previous studies indicate that telephone-based self-reports of smoking abstinence can overestimate cessation rates by approximately 5–10%.^21,22^ However, this bias would likely affect both study arms equally. As participants were recruited from a single smoking cessation center, findings may not be generalizable to other clinical settings or populations. Although this study was not primarily designed to compare the efficacy of different smoking cessation medications, we report an interesting finding regarding their comparable effectiveness in a real-world clinical setting. This observation of similar effectiveness among anti-smoking drugs likely arises from the fact that clinicians tailor drug selection for each patient based on individualized clinical reasoning. This process considers a multitude of variables, including the patient's clinical and smoking history, motivation levels, and prior quitting attempts, enabling them to choose the most appropriate medication for that specific individual at that particular moment. While aiming to optimize individual outcomes, this context-dependent drug selection may contribute to a leveling effect when analyzing effectiveness at a group level, potentially masking inherent differences observed in controlled trial settings. Furthermore, genetic information represents another valuable parameter that can further refine therapy selection for the doctor and the patient, moving beyond a one-size-fits-all approach. An interesting finding emerges from the comparison between cytisine and varenicline, two drugs that share the same mechanism of action. Indeed, at all four follow-up times, there were no significant differences in the numbers of individuals who quit smoking between those treated with these two drugs. This is a comforting result, considering that varenicline has been withdrawn from the market in our country and replaced by cytisine. These results should be verified through a randomized controlled equivalence trial. So far, contrasting results have been reported in this regard^23^ and, therefore, further randomized placebo-controlled clinical trials should be helpful to investigate this issue better.

## Conclusion

In conclusion, the outcomes of this preliminary investigation indicate that awareness of the *CHRNA5* rs503464 genotype does not manifest a statistically significant impact on the probability of success or failure in an individual's smoking cessation trajectory. As a corollary, the integration of genotype communication before therapeutic agent selection and initiation warrants consideration. Nonetheless, further rigorous investigation is imperative to elucidate the biological underpinnings of potential interactions between pharmacological interventions and genetic profiles, and their respective contributions to smoking cessation outcomes. From our perspective, a substantive requirement persists for more extensive pharmacogenomic analyses about smoking cessation therapies. Notwithstanding this necessity for continued inquiry, the current findings delineate prospective future directions increasingly aligned with implementing personalized medicine paradigms in the context of smoking cessation.

## Supplemental Material

sj-docx-1-tmj-10.1177_03008916251408279 – Supplemental material for The clinical usefulness of knowing CHRNA5 polymorphism genotype: paving the way for personalized therapy Supplemental material, sj-docx-1-tmj-10.1177_03008916251408279 for The clinical usefulness of knowing CHRNA5 polymorphism genotype: paving the way for personalized therapy by Francesca Colombo, Chiara Veronese, Elena Munarini, Cinzia Paolino, Davide Maspero, Nunzia Mangano, Martina Esposito, Francesca Minnai, Sara Noci, Marta Giussani, Daniele Morelli, Elisa Cardani, Alessandro Esposito, Gaia Giulia Angela Sacco, Debora Spitaleri and Roberto Boffi in Tumori Journal

sj-png-2-tmj-10.1177_03008916251408279 – Supplemental material for The clinical usefulness of knowing CHRNA5 polymorphism genotype: paving the way for personalized therapy Supplemental material, sj-png-2-tmj-10.1177_03008916251408279 for The clinical usefulness of knowing CHRNA5 polymorphism genotype: paving the way for personalized therapy by Francesca Colombo, Chiara Veronese, Elena Munarini, Cinzia Paolino, Davide Maspero, Nunzia Mangano, Martina Esposito, Francesca Minnai, Sara Noci, Marta Giussani, Daniele Morelli, Elisa Cardani, Alessandro Esposito, Gaia Giulia Angela Sacco, Debora Spitaleri and Roberto Boffi in Tumori Journal

sj-xlsx-3-tmj-10.1177_03008916251408279 – Supplemental material for The clinical usefulness of knowing CHRNA5 polymorphism genotype: paving the way for personalized therapy Supplemental material, sj-xlsx-3-tmj-10.1177_03008916251408279 for The clinical usefulness of knowing CHRNA5 polymorphism genotype: paving the way for personalized therapy by Francesca Colombo, Chiara Veronese, Elena Munarini, Cinzia Paolino, Davide Maspero, Nunzia Mangano, Martina Esposito, Francesca Minnai, Sara Noci, Marta Giussani, Daniele Morelli, Elisa Cardani, Alessandro Esposito, Gaia Giulia Angela Sacco, Debora Spitaleri and Roberto Boffi in Tumori Journal
