# Supplementary material for: The clinical usefulness of knowing CHRNA5 polymorphism genotype: paving the way for personalized therapy
Source: Tumori. 2026 Mar 10;112(3):221–30. doi: 10.1177/03008916251408279 (PMC13250266; doi:10.1177/03008916251408279)
Supplement: sj-docx-1-tmj-10.1177_03008916251408279 – Supplemental material for The clinical usefulness of knowing CHRNA5 polymorphism genotype: paving the way for personalized therapy [file sj-docx-1-tmj-10.1177_03008916251408279.docx]

***Supplementary 1.*** *Disclosure of Genetic Information*

For the randomly assigned study you agreed to participate in, you are in the group of those who can be informed about your genetic information. With respect to greater or lesser difficulty quitting smoking, you were found to be in the lower/higher risk group.
