# Supplementary figures and images for: The clinical usefulness of knowing CHRNA5 polymorphism genotype: paving the way for personalized therapy
Source: Tumori. 2026 Mar 10;112(3):221–30. doi: 10.1177/03008916251408279 (PMC13250266; doi:10.1177/03008916251408279)

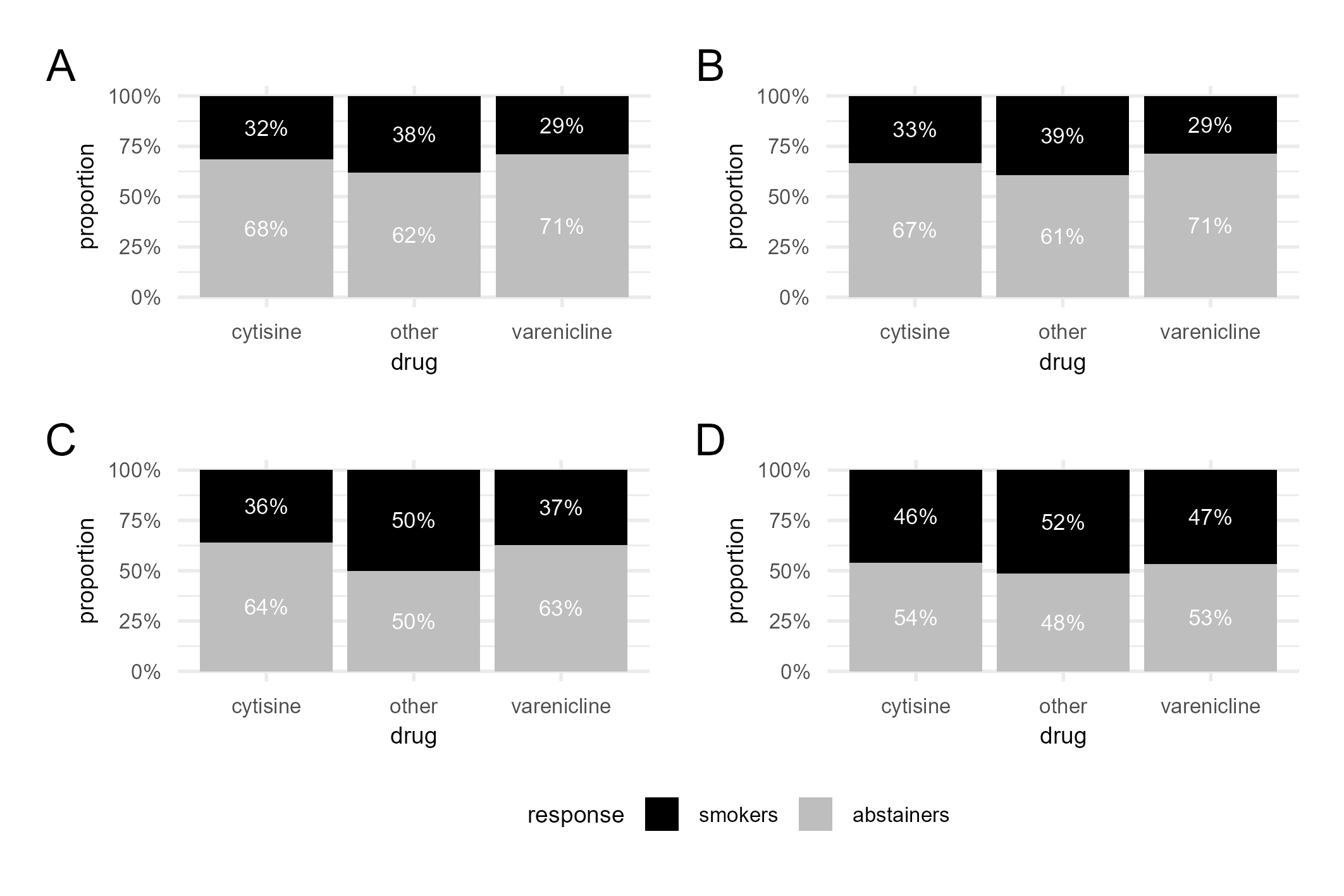

Supplement: sj-png-2-tmj-10.1177_03008916251408279 – Supplemental material for The clinical usefulness of knowing CHRNA5 polymorphism genotype: paving the way for personalized therapy [file sj-png-2-tmj-10.1177_03008916251408279.png]
